# Secondary Analysis of the NCI-60 Whole Exome Sequencing Data Indicates Significant Presence of *Propionibacterium acnes* Genomic Material in Leukemia (RPMI-8226) and Central Nervous System (SF-295, SF-539, and SNB-19) Cell Lines

**DOI:** 10.1371/journal.pone.0127799

**Published:** 2015-06-03

**Authors:** Mark Rojas, Georgiy Golovko, Kamil Khanipov, Levent Albayrak, Sergei Chumakov, B. Montgomery Pettitt, Alex Y. Strongin, Yuriy Fofanov

**Affiliations:** 1 Department of Pharmacology and Toxicology, University of Texas Medical Branch, Galveston, Texas, United States of America; 2 Department of Biochemistry and Molecular Biology, University of Texas Medical Branch, Galveston, Texas, United States of America; 3 Sealy Center for Structural Biology, University of Texas Medical Branch, Galveston, Texas, United States of America; 4 Department of Physics, University of Guadalajara, Guadalajara, Jalisco, Mexico; 5 Inflammatory and Infectious Disease Center/Cancer Research Center, Sanford-Burnham Medical Research Institute, La Jolla, California, United States of America; University of Ulster, UNITED KINGDOM

## Abstract

The NCI-60 human tumor cell line panel has been used in a broad range of cancer research over the last two decades. A landmark 2013 whole exome sequencing study of this panel added an exceptional new resource for cancer biologists. The complementary analysis of the sequencing data produced by this study suggests the presence of *Propionibacterium acnes* genomic sequences in almost half of the datasets, with the highest abundance in the leukemia (RPMI-8226) and central nervous system (SF-295, SF-539, and SNB-19) cell lines. While the origin of these contaminating bacterial sequences remains to be determined, observed results suggest that computational control for the presence of microbial genomic material is a necessary step in the analysis of the high throughput sequencing (HTS) data.

## Introduction

Since the late 1980s, the National Cancer Institute's NCI-60 panel representing nine tumor types (breast, central nervous system, colon, leukemia, lung, melanoma, ovarian, prostate, and renal) has been one of the most highly characterized sets of reference cell lines [1], and is considered to be a valuable public resource for cancer research and drug discovery. Considering that many laboratories world-wide rely on NCI-60 panel cell lines to identify anticancer agents, the quality of the cells and associated data is a priority in cell and cancer biology. However, some level of bacterial and viral genomic material has been observed in a variety of high throughput sequencing datasets presumed to be sterile [2]. Several attempts to perform secondary analysis of large, publically available, HTS data have also been successful in identifying traces of viral genomic material [3–6]. To date, DNA fingerprinting has been shown to be effective for validation of cell identification and origin, but it did not provide any information related to potential microbial contamination [7]. Here, we employed recently developed computational tools for the detection of viral and bacterial pathogens in clinical samples to re-analyze the raw sequencing data of the NCI-60 panel in an effort to rule out the presence of microbial contamination.

## Materials and Methods

### NCI-60 Dataset

The whole exome sequencing (WES) dataset for the NCI-60 panel was originally published as supplementary material by *Abaan et al*. [8]. In September 2013, sixty-one NCI-60 WES files were downloaded in BAM format (a compressed binary version of a sequence alignment/map file) currently available at http://watson.nci.nih.gov/projects/nci60/wes/BAMS/. The files were then converted to the FASTQ format using bam2fastq 1.1.0 (HudsonAlpha: http://www.hudsonalpha.org/gsl/information/software/bam2fastq.) To ensure that the high quality reads alone were used in the analysis, all nucleotides with a quality score below 10 were treated as unknown and replaced with the unknown nucleotide symbol “N”.

### Gene Cluster Database and Reads Mapping Algorithm

To determine the presence and identity of microbial contaminants, the sequencing data was tested against a collection of 13,774 complete bacterial, viral, plasmid, phage, and fungal reference genome sequences, which included all reference genomes available from the NCBI Reference Sequence (RefSeq) collection as of November 2013. The complete list of the reference genomes used in the analysis can be found on the supplementary website (http://scsb.utmb.edu/faculty/fofanov/nci60.asp). GenBank records for each complete reference genome were parsed using, as reference, their genome accession numbers to collect every coding sequence (CDS), resulting in 9,352,435 gene sequences. To eliminate redundancy, these sequences were organized into 6,064,751 clusters containing sequences with a minimal level of similarity of 95%, such that only a single representative sequence for each cluster could be used in the sequence search and alignment.

In order to map (align) a large number (greater than 7 billion) of subsequences (reads) to over six million reference cluster sequences totaling more than 5Gb in length, we used a reference-based mapping approach, developed in-house, where all reads are stored in a search efficient data structure (i.e., sorted array incorporated with modified suffix tree). In contrast to traditional read-by-read mapping methods (e.g., BWA[9], Bowtie[10]) where each read is searched against reference sequence(s), this method can perform a search for any number of reference sequences (where each subsequence of reads length is acquired from the reference sequence) in a very short time: it takes less than 30 minutes utilizing a single CPU to map approximately 30 million reads to 6 million cluster sequences. It is also important to mention that if only perfect match alignments are considered, the time complexity is not dependent on the number of reads in the dataset. As a result, alignment files, representing nucleotide and/or reads coverage across reference sequences were generated with a relatively fast turnaround time.

### Analysis

The presence of microbial genomic material was determined using a two-step approach. First, to identify potential candidates for each of the 61 samples, 32-base long subsequences were extracted from the original 80-base long reads with no overlap: first subsequence was defined as left most subsequence for which each nucleotide is known and passed the quality threshold, the second subsequence was defined as the left most subsequence starting from position following the end of the first subsequence for which each nucleotide is known and passed the quality threshold. The 32-base long subsequences were then mapped with no mismatches to all 6,064,751 cluster sequences resulting in a “cluster profile” for each sample (clusters of less than 50 bases in size were not considered). The logic behind the choice of using 32-base long unpaired subsequences (when original reads were 80 bases paired) is a compromise between time complexity, sensitivity, and specificity of the detection procedure: 32-mers are long enough to be highly specific and not “interfere” (appear simultaneously in human and bacteria) with the background. For a minority of subsequences, where their frequency of appearance in human and bacterial genomes are known to be significantly lower and/or higher than expected for random sequences [11–13], the appearance of the majority of subsequences can still be described using the random model [14,15]. For example, the expected number of sequences in the set of 30 million reads (average number of reads in each NCI-60 dataset) to be found present in human and 5 Mb long bacterial genomes simultaneously is 1.63 10^–5^ for 32-mers and 2.05 10^–34^ for 80-mers (Appendix 1 in S2 Text).

It is also obvious that in order for a longer (80 bases) sequence to be perfectly aligned to the reference, its shorter (32 bases) subsequences must also be aligned to the reference. In the presented work, this approach (mapping 32-mers first) was used to rule out microorganisms’ genomic sequences that are not present in the samples. In the course of this step of the analysis, original reads were disassembled into non-overlapping 32-base long subsequences containing only high quality nucleotides (no “N” was allowed). Low complexity subsequences with a single nucleotide domination (i.e., a single nucleotide type represented in more than 90% of the positions in subsequence) and/or limited representation (i.e., a single nucleotide type represented in less than 5% of the positions in a subsequence) were also excluded. The total number of the original reads acquired from the BAM files and statistics for the selected subsequences are available in Section A in S1 Text. Clusters were considered present if they had at least one subsequence mapped to them with no mismatches. The cluster profiles (list of presence in sample clusters) of each of the 61 samples were then compared with the cluster composition of each of the 13,774 reference genomes. Genomes with over 5% of their clusters present in the sample cluster profile were selected as candidates for the second step of the analysis in which both 32-base and 80-base long subsequences were mapped to the complete sequences of the candidate reference genomes.

## Results and Discussion

**Fig 1:**
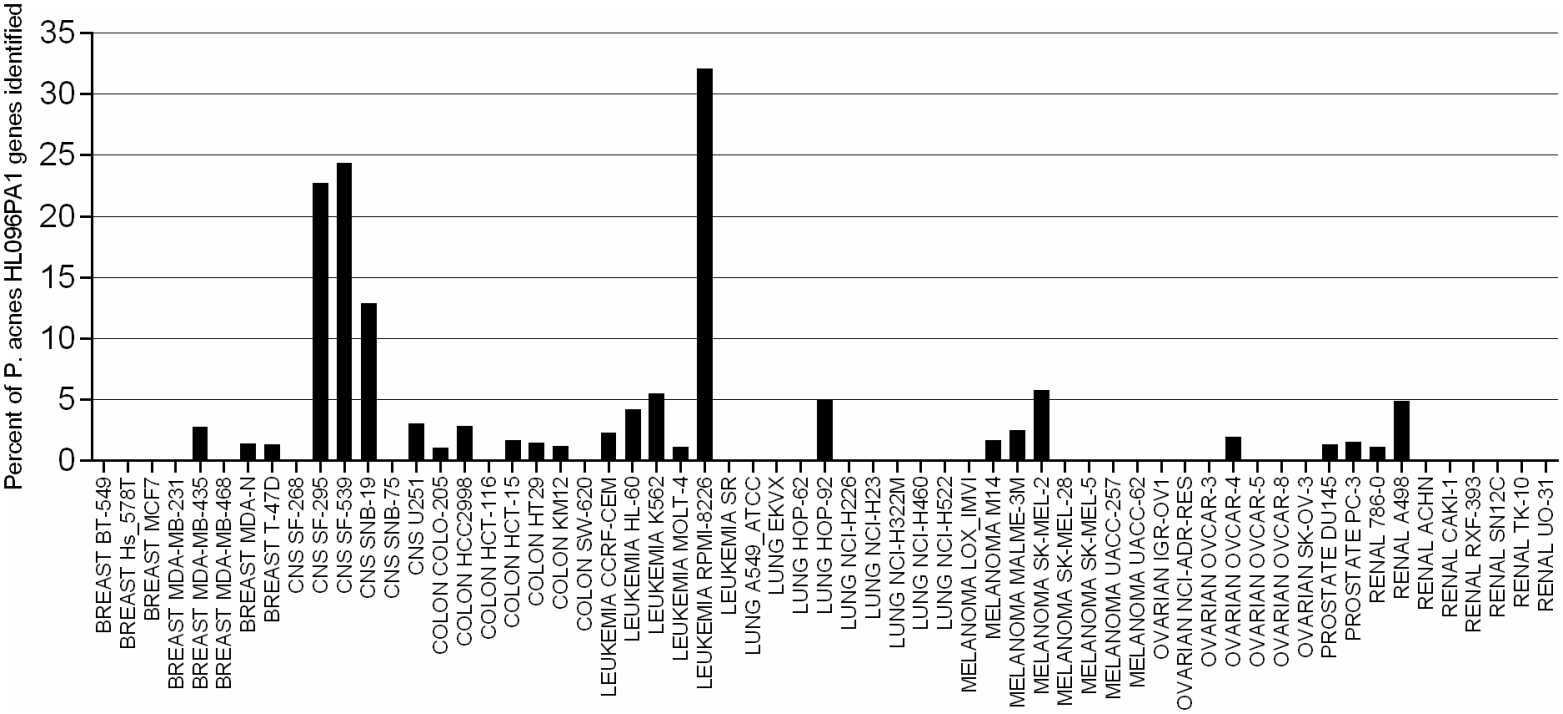
Percent of P. *acnes* HL096PA1 genes identified. The percent of genes identified out of 2,175 for P. *acnes* HL096PA1 (NC_021085.1) across all 61 NCI-60 datasets. A gene was considered identified if one or more reads (32-base long subsequences) mapped with no genomic variation (zero mismatch).

From the entire collection of bacterial, viral, phage, fungal, and plasmid reference genome sequences used in the analysis, *Propionibacterium acnes* alone exhibited a significant presence in the NCI-60 cells (Fig 1 and Section B in S1 Text). The leukemia (RPMI-8226) and central nervous system (SF-295, SF-539, and SNB-19) samples exhibited the highest amount of *P*. *acnes* genomic material ranging from 800 subsequences (reads) and 290 genes in SNB-19 to over 3000 subsequences and more than 730 genes in RPMI-8226 samples.

The complete list of sequences mapped to *P*. *acnes* genomes for each of the four cell samples is available on the supplementary website. Virtually all identified 32-base long *P*. *acnes* subsequences are uniquely (in single copy) present in *P*. *acnes* genomes (Section C in S1 Text). The nucleotide BLAST search version 2.2.28+ with word length 11 (all other parameters remained set at default) for each identified 32-mer against the human genome (February 2014, Annotation Release 106) and an exhaustive search (no mismatches allowed) against the human reference genome (build 37.2) confirmed that none of these sequences originated from the human genome.

A simple model can be proposed (Appendix 2 in S2 Text) to estimate the original “proportion” of the bacteria in the exome enriched samples. For 5% of 20-fold enriched human genome, it predicted that for 2,000 of the 30,000,000 subsequences (reads), the original proportion of bacteria in the sample is approximately one bacterium per six human cells, which for many infectious agents would be considered a high rate of the infection. It is also important to mention that there is virtually no intersection between the sets of reads originated from different samples and those mapped to *P*. *acnes*, which is expected under the assumption that reads are coming from random locations of the microbial genome.

**Fig 2:**
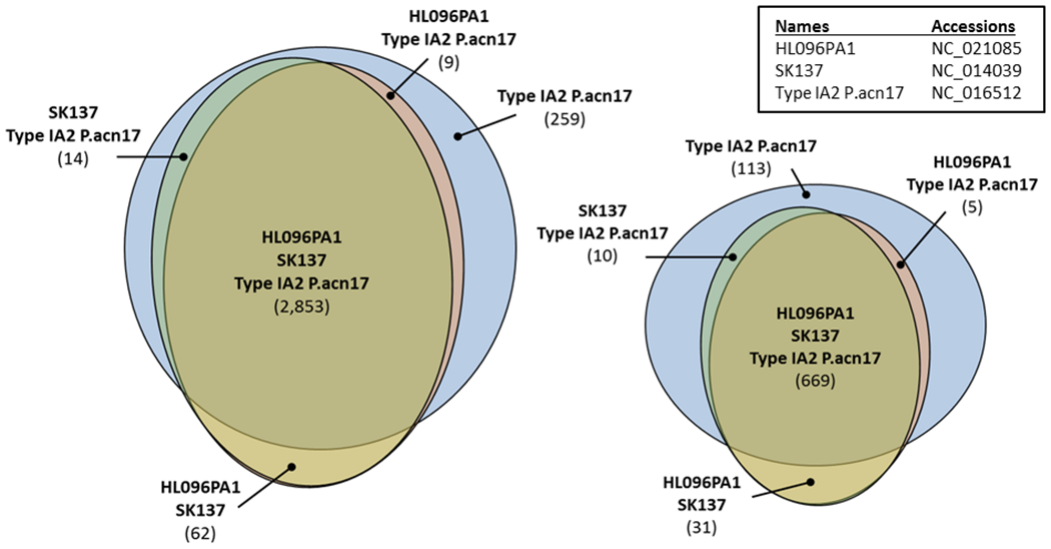
Comparison of unique sequences and subsequences for RPMI 8226. Venn diagrams showing the difference between the number of unique 32-base long subsequences (left) and 80-base long sequences (right) from leukemia RPMI 8226 cell line sample that mapped to (with perfect match) and shared by three reference P. *acnes* genomes (HL096PA1, SK137, and Type IA2 P.acn17).

To confirm the mapping results for the 32-base long subsequences indicating the presence of bacterial genomic sequences in the dataset, the original 80-base long reads from all four datasets were mapped (with perfect match) to the reference *P*. *acnes*. The overall number of 80-base long reads that mapped to *P*. *acnes* appears about five times lower than for 32-base long reads (Fig 2) reflecting the facts that: a) not all high quality 80-mers were mapped to the reference genome(s) with perfect match, where a single or even both 32-base long subsequences were mapped; and b) some of the 80-mers were excluded from consideration because they did not contain high quality nucleotides across the entire sequence, yet still contained high quality 32-mers which were used during the first step of the analysis.

**Fig 3:**
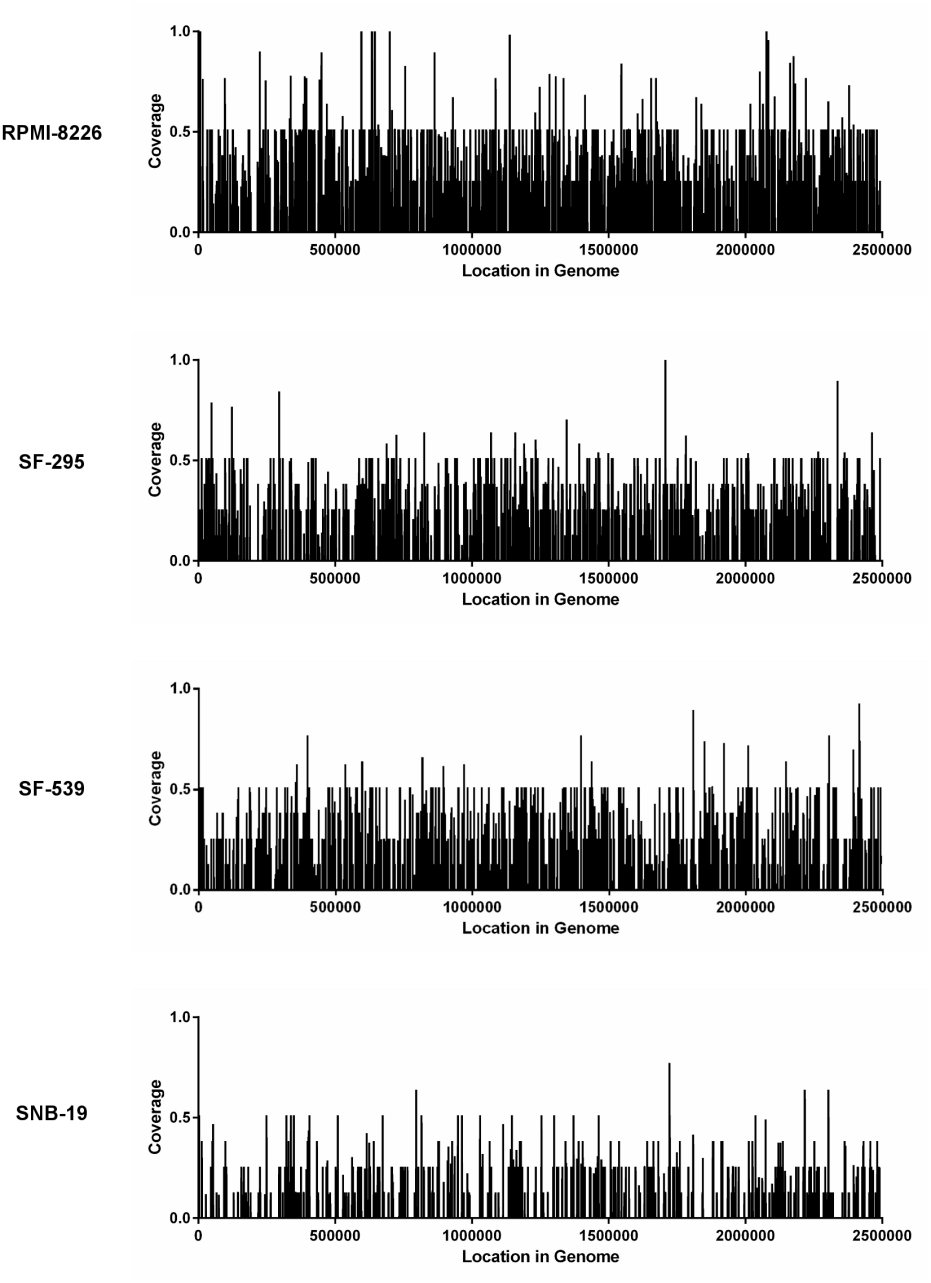
Average windowed nucleotide coverage for P. *acnes* HL096PA1. Average window-by-window nucleotide coverage (window size = 250 bases) of the P. *acnes* HL096PA1 (NC_021085.1) genome by 32-base long subsequences acquired from leukemia (RPMI-8226) and central nervous system (SF-295, SF-539, and SNB-19) sequencing datasets. The average nucleotide coverage per window is equal to the total number of reads that map (cover) each position within the given window divided by the window size (250).

Given that more than a dozen NCI-60 cell lines did not exhibit any detectable *P*. *acnes* genomic material, it is nearly impossible to conclude that the presence of the microbial subsequences detected in the samples is an artifact caused by the presence of 32-base long subsequences in the human genome that are identical to bacteria. The fact that out of over 13,000 bacterial, viral, phage, fungal and plasmid genomes, *P*. *acnes* alone was identified in the four cell types, leads to the conclusion that the results are highly specific for this particular microorganism. This conclusion is also supported by the fact that the identified sequencing reads are evenly distributed along the entire *P*. *acnes* genome (Fig 3 and Section E in S1 Text), rather than clustered in some highly conserved sequence regions such as ribosomal RNAs. It is also important to emphasize that if any other *P*. *acnes* bacteria were present in the sample, but not detected because its genome was not present among the reference genomes used in the analysis, then the coverage for the three P. *acnes* rRNA operons would be expected to be higher due to a high level of sequence similarities between all rRNA sequences. Because such increase of coverage was not observed in our analysis, this leads us to conclude that P. *acnes* is the only bacterial present in significant abundance. (Fig 3 and Section E in S1 Text).

It is necessary to emphasize that the presence of *P*. *acnes* in the samples cannot affect the DNA (exome) profile of the cells, which leads us to believe that regardless of the source of bacterial genomic material our observations are not questioning the conclusion and results of the original study [8]. However, considering its association to inflammation, the presence of these bacteria in cell lines can introduce significant bias in gene expression and methylation patterns.

## Conclusion

The presented data imply that there is a microbial contamination of either the highly valuable and widely used cell lines or, probably, a contamination was introduced in the course of DNA processing and/or sequencing procedures. While the true origin of the *P*. *acnes* genomic sequences in the NCI-60 sequencing data remains to be determined, we believe that any sequence alignment software (e.g. BWA, Bowtie) is capable to identify the presence of “hidden” microbial contamination in cell/tissue samples; however, a not too time-consuming computational analysis approach such as the one we employed should be used as a quality control step in any type of analysis of HTS data regardless of the focus of the study.

## Supporting Information

S1 Text Supplemental Document. (DOCX)

S2 Text Appendix. (DOCX)
