# Supplementary material for: Secondary Analysis of the NCI-60 Whole Exome Sequencing Data Indicates Significant Presence of Propionibacterium acnes Genomic Material in Leukemia (RPMI-8226) and Central Nervous System (SF-295, SF-539, and SNB-19) Cell Lines
Source: PLoS One. 2015 Jun 3;10(6):e0127799. doi: 10.1371/journal.pone.0127799 (PMC4454691; doi:10.1371/journal.pone.0127799)
Supplement: S1 Text — (DOCX) [file pone.0127799.s001.docx]

**Secondary analysis of the NCI-60 whole exome sequencing data indicates *Propionibacterium acnes* genomic material in Leukemia (RPMI-8226) and Central Nervous System (SF-295, SF-539, and SNB-19) cell lines**

**(Supplementary Materials)**

Mark Rojas^1,3*^, Georgiy Golovko^1,3^, Kamil Khanipov^1,3^, Levent Albayrak^1,3^, Sergei Chumakov^4^, B. Montgomery Pettitt^1,2,3^, Alex Y. Strongin^5^, Yuriy Fofanov^1,3^

^1^Department of Pharmacology and Toxicology, University of Texas Medical Branch, Galveston, Texas, United States of America

^2^Department of Biochemistry and Molecular Biology, University of Texas Medical Branch, Galveston, Texas, United States of America

^3^Sealy Center for Structural Biology, University of Texas Medical Branch, Galveston, Texas, United States of America

^4^Department of Physics, University of Guadalajara, Guadalajara, Jalisco, Mexico

^5^Inflammatory and Infectious Disease Center/Cancer Research Center, Sanford-Burnham Medical Research Institute, La Jolla, California, United States of America

* Corresponding author

E-mail: [mmrojas@utmb.edu](mailto:mmrojas@utmb.edu) (MR)

1. **Supplementary Table 1:** Raw reads and selected 32-base long subsequences used in the analysis.

| **Cell Panel** | **Cell Line** | **FASTQ file format** | | **AS file format** | |
| --- | --- | --- | --- | --- | --- |
|  |  | **Total Reads** | **Unique Reads** | **Total Reads** | **Unique Reads** |
| BREAST | BT-549 | 38,080,816 | 34,480,896 | 61,028,670 | 41,664,495 |
| BREAST | Hs_578T | 135,606,520 | 132,610,786 | 330,399,897 | 153,020,124 |
| BREAST | MCF7 | 148,269,804 | 143,828,451 | 356,183,530 | 154,902,176 |
| BREAST | MDA-MB-231 | 42,447,437 | 39,530,895 | 52,351,812 | 39,873,926 |
| BREAST | MDA-MB-468 | 42,530,864 | 38,717,976 | 64,137,534 | 43,847,383 |
| BREAST | T-47D | 138,270,424 | 133,563,797 | 344,799,216 | 147,408,204 |
| CNS | SF-268 | 90,657,644 | 79,040,139 | 134,508,319 | 83,399,935 |
| CNS | SF-295 | 25,165,810 | 22,999,288 | 41,096,643 | 30,348,365 |
| CNS | SF-539 | 70,718,308 | 62,275,655 | 116,292,683 | 73,907,277 |
| CNS | SNB-19 | 40,953,166 | 37,851,773 | 59,707,065 | 43,225,465 |
| CNS | SNB-75 | 38,486,420 | 35,634,387 | 57,900,362 | 42,371,367 |
| CNS | U251 | 185,958,934 | 152,042,860 | 285,604,256 | 149,516,556 |
| COLON | COLO-205 | 78,518,070 | 68,446,974 | 125,902,695 | 78,396,215 |
| COLON | HCC2998 | 96,867,544 | 85,555,144 | 133,199,701 | 86,399,940 |
| COLON | HCT-116 | 43,117,490 | 37,232,153 | 75,453,380 | 51,089,764 |
| COLON | HCT-15 | 47,383,518 | 42,671,415 | 84,550,780 | 58,422,007 |
| COLON | HT29 | 207,375,774 | 201,928,294 | 331,954,113 | 152,337,527 |
| COLON | KM12 | 55,609,626 | 51,071,227 | 90,456,005 | 61,756,968 |
| COLON | SW-620 | 25,432,444 | 22,660,844 | 63,627,028 | 40,411,139 |
| LEUKEMIA | CCRF-CEM | 69,225,682 | 60,631,598 | 113,721,323 | 77,333,720 |
| LEUKEMIA | HL-60 | 66,983,794 | 58,934,050 | 113,748,718 | 76,892,602 |
| LEUKEMIA | K562 | 73,138,506 | 62,617,219 | 119,340,373 | 77,078,371 |
| LEUKEMIA | MOLT-4 | 88,792,816 | 82,026,282 | 85,906,794 | 52,842,060 |
| LEUKEMIA | RPMI-8226 | 42,758,234 | 35,981,454 | 75,901,587 | 49,646,517 |
| LEUKEMIA | SR | 42,617,400 | 36,431,273 | 75,136,347 | 50,524,157 |
| LUNG | A549_ATCC | 39,512,536 | 35,120,889 | 65,816,023 | 48,819,509 |
| LUNG | EKVX | 211,112,798 | 185,252,957 | 179,948,037 | 87,989,813 |
| LUNG | HOP-62 | 82,374,760 | 64,255,175 | 156,137,303 | 75,270,928 |
| LUNG | HOP-92 | 44,327,612 | 38,687,056 | 78,652,540 | 55,760,474 |
| LUNG | NCI-H226 | 51,950,210 | 43,272,444 | 66,141,422 | 39,502,210 |
| LUNG | NCI-H23 | 25,525,656 | 22,721,961 | 64,293,358 | 41,435,275 |
| LUNG | NCI-H322M | 25,189,256 | 22,116,997 | 43,689,734 | 30,652,058 |
| LUNG | NCI-H460 | 78,658,552 | 66,514,818 | 131,404,165 | 66,785,430 |
| LUNG | NCI-H522 | 36,920,284 | 32,126,709 | 64,092,332 | 44,820,304 |
| MELANOMA | LOX_IMVI | 41,771,180 | 34,091,024 | 104,127,096 | 57,502,106 |
| MELANOMA | M14 | 96,281,370 | 93,896,152 | 205,019,770 | 117,876,720 |
| MELANOMA | MALME-3M | 85,384,460 | 76,419,255 | 143,496,681 | 89,755,731 |
| MELANOMA | MDA-MB-435 | 73,996,784 | 63,343,666 | 126,823,239 | 81,976,229 |
| MELANOMA | MDA-N | 104,431,958 | 95,933,748 | 137,135,726 | 89,592,739 |
| MELANOMA | SK-MEL-2 | 88,839,478 | 66,677,983 | 80,117,390 | 56,671,218 |
| MELANOMA | SK-MEL-28 | 45,431,858 | 39,410,730 | 125,195,462 | 60,970,472 |
| MELANOMA | SK-MEL-5 | 49,506,876 | 42,175,317 | 61,971,390 | 37,913,504 |
| MELANOMA | UACC-257 | 25,090,246 | 21,877,090 | 63,871,774 | 40,590,743 |
| MELANOMA | UACC-62 | 74,006,108 | 73,012,274 | 193,823,380 | 94,309,007 |
| OVARIAN | IGR-OV1 | 26,038,252 | 24,129,976 | 27,412,121 | 20,786,726 |
| OVARIAN | NCI-ADR-RES | 51,518,878 | 44,221,797 | 64,304,966 | 39,900,007 |
| OVARIAN | OVCAR-3 | 35,390,492 | 30,660,719 | 64,985,283 | 43,707,372 |
| OVARIAN | OVCAR-4 | 167,021,852 | 154,408,423 | 167,193,137 | 101,668,155 |
| OVARIAN | OVCAR-5 | 83,480,938 | 70,514,259 | 144,910,892 | 81,123,390 |
| OVARIAN | OVCAR-8 | 24,235,220 | 22,803,381 | 38,816,947 | 30,598,946 |
| OVARIAN | SK-OV-3 | 50,169,864 | 42,476,726 | 64,513,731 | 39,993,098 |
| PROSTATE | DU145 | 25,170,816 | 21,542,002 | 37,537,962 | 25,648,737 |
| PROSTATE | PC-3 | 59,756,978 | 45,680,137 | 89,137,925 | 47,092,990 |
| RENAL | 786-0 | 88,109,656 | 74,440,045 | 152,294,326 | 85,664,970 |
| RENAL | A498 | 68,709,374 | 59,722,021 | 119,414,385 | 79,133,596 |
| RENAL | ACHN | 88,260,518 | 77,638,365 | 135,980,131 | 88,896,902 |
| RENAL | CAKI-1 | 24,355,992 | 22,923,700 | 39,267,965 | 31,038,129 |
| RENAL | RXF-393 | 79,249,984 | 71,399,820 | 131,970,994 | 83,581,672 |
| RENAL | SN12C | 28,151,366 | 25,150,592 | 47,002,670 | 33,664,677 |
| RENAL | TK-10 | 85,606,008 | 62,580,714 | 150,350,301 | 73,432,609 |
| RENAL | UO-31 | 44,040,184 | 34,450,325 | 113,002,972 | 61,893,254 |

1. **Supplementary Table 2:** Number of genes present and reads mapped to the *P. acnes* genomes from the leukemia (RPMI-8226) and central nervous system (SF-295, SF-539, and SNB-19) cell lines.

| Accession | Genome | Number of genes (clusters) longer than 50bp used in analysis | Human Leukemia cell line RPMI-8226 | | Human CNS cell line  SF-295 | | Human CNS cell line  SF-539 | | Human CNS cell line  SNB-19 | |
| --- | --- | --- | --- | --- | --- | --- | --- | --- | --- | --- |
|  |  |  | Number of Genes Identified | Number of Reads Mapped | Number of Genes Identified | Number of Reads Mapped | Number of Genes Identified | Number of Reads Mapped | Number of Genes Identified | Number of Reads Mapped |
| NC_018707.1 | P. acnes C1 | 2359 | 731 | 2949 | 502 | 1793 | 543 | 1833 | 282 | 791 |
| NC_021085.1 | P. acnes HL096PA1 | 2175 | 698 | 2924 | 495 | 1778 | 530 | 1840 | 281 | 779 |
| NC_017550.1 | P. acnes ATCC 11828 | 2259 | 627 | 1622 | 437 | 987 | 493 | 1130 | 252 | 502 |
| NC_016516.1 | P. acnes TypeIA2 P.acn33 | 2233 | 730 | 3163 | 506 | 1731 | 546 | 1887 | 284 | 825 |
| NC_016512.1 | P. acnes TypeIA2 P.acn17 | 2263 | 735 | 3135 | 507 | 1749 | 542 | 1866 | 285 | 812 |
| NC_017534.1 | P. acnes 266 | 2345 | 737 | 2957 | 503 | 1808 | 547 | 1853 | 292 | 803 |
| NC_016511.1 | P. acnes TypeIA2 P.acn31 | 2244 | 728 | 3131 | 503 | 1755 | 546 | 1874 | 286 | 823 |
| NC_014039.1 | P. acnes SK137 | 2352 | 740 | 2929 | 516 | 1776 | 557 | 1839 | 288 | 784 |
| NC_017535.1 | P. acnes 6609 | 2348 | 708 | 2708 | 497 | 1703 | 525 | 1742 | 285 | 750 |
| NC_006085.1 | P. acnes KPA171202 | 2297 | 717 | 2701 | 505 | 1697 | 537 | 1739 | 287 | 747 |

1. **Supplementary Table 3.** 32-mer sequences present/mapped to the *P. acnes* genomes from the leukemia (RPMI-8226) and central nervous system (SF-295, SF-539, and SNB-19) cell lines.

| Combined Table  (*P. acnes* samples) | Copy number 1  Mapped/Present | Copy number 2  Mapped/Present | Copy number 3  Mapped/Present | Copy number 4  Mapped/Present | Copy number 5  Mapped/Present | Copy number 6  Mapped/Present |
| --- | --- | --- | --- | --- | --- | --- |
| HL096PA1 | 2878 / 4929348 | 2 / 4251 | 44 / 16690 | 0 / 36 | 0 / 10 | 0 / 34 |
| TypeIA2 | 3077 / 4989892 | 6 / 4745 | 52 / 15426 | 0 / 0 | 0 / 0 | 0 / 8 |
| SK137 | 2883 / 4931630 | 2 / 4809 | 44 / 16418 | 0 / 14 | 0 / 0 | 0 / 8 |
| 266 | 2908 / 4931646 | 5 / 4279 | 44 / 16254 | 0 / 4 | 0 / 0 | 0 / 8 |

1. **Supplementary Table 4.** Number of 80-base long reads from leukemia (RPMI-8226) and central nervous system (SF-295, SF-539, and SNB-19) cell lines dataset mapped to *P. acnes* genomes

| **Combined Table** | ***RPMI-8226*** | ***SF-295*** | ***SF-539*** | ***SNB-19*** |
| --- | --- | --- | --- | --- |
| P. acnes HL096PA1 | 705 | 266 | 272 | 59 |
| P. acnes TypeIA2 | 797 | 259 | 281 | 70 |
| P. acnes SK137 | 710 | 266 | 275 | 62 |
| P. acnes 266 | 715 | 280 | 280 | 63 |

1. **Supplementary Figure 1.** An average window-by-window nucleotide coverage of *P.acnes* HL096PA1 (NC_021085.1) genome by the 32-base long subsequences acquired from all 52 NCI-60 datasets. The remaining 9 datasets did not show any presence of *P.acnes*. The window size = 250 bases.
2. HUMAN BREAST CARCINOMA CELL LINE MCF7
3. HUMAN BREAST CARCINOMA CELL LINE MDA-MB-231
4. HUMAN BREAST CARCINOMA CELL LINE MDA-MB-468
5. HUMAN BREAST CARCINOMA CELL LINE T-47D
6. HUMAN CENTRAL NERVOUS SYSTEM CELL LINE SF-268
7. HUMAN CENTRAL NERVOUS SYSTEM CELL LINE SF-295
8. HUMAN CENTRAL NERVOUS SYSTEM CELL LINE SF-539
9. HUMAN CENTRAL NERVOUS SYSTEM CELL LINE SNB-19
10. HUMAN CENTRAL NERVOUS SYSTEM CELL LINE SNB-75
11. HUMAN CENTRAL NERVOUS SYSTEM CELL LINE U251
12. HUMAN COLON CELL LINE COLO-205
13. HUMAN COLON CELL LINE HCC2998
14. HUMAN COLON CELL LINE HCT-116
15. HUMAN COLON CELL LINE HCT-15
16. HUMAN COLON CELL LINE, GII HT29
17. HUMAN COLON CELL LINE KM12
18. HUMAN COLON CELL LINE SW-620
19. HUMAN LEUKEMIA CELL LINE CCRF-CEM
20. HUMAN LEUKEMIA CELL LINE HL-60
21. HUMAN LEUKEMIA CELL LINE K562
22. HUMAN LEUKEMIA CELL LINE MOLT-4
23. HUMAN LEUKEMIA CELL LINE RPMI-8226
24. HUMAN LEUKEMIA CELL LINE SR
25. HUMAN LUNG CELL LINE A549_ATCC
26. HUMAN LUNG CELL LINE EKVX
27. HUMAN LUNG CELL LINE HOP-62
28. HUMAN LUNG CELL LINE HOP-92
29. HUMAN LUNG CELL LINE NCI-H226
30. HUMAN LUNG CELL LINE NCI-H322M
31. HUMAN LUNG CELL LINE NCI-H522
32. HUMAN MELANOMA CARCINOMA CELL LINE MDA-MB-435
33. HUMAN MELANOMA CARCINOMA CELL LINE MDA-N
34. HUMAN MELANOMA CELL LINE M14
35. HUMAN MELANOMA CELL LINE MALME-3M
36. HUMAN MELANOMA CELL LINE SK-MEL-2
37. HUMAN MELANOMA CELL LINE SK-MEL-28
38. HUMAN MELANOMA CELL LINE SK-MEL-5
39. HUMAN MELANOMA CELL LINE UACC-257
40. HUMAN MELANOMA CELL LINE UACC-62
41. HUMAN OVARIAN CELL LINE NCI-ADR-RES
42. HUMAN OVARIAN CELL LINE OVCAR-4
43. HUMAN OVARIAN CELL LINE OVCAR-5
44. HUMAN OVARIAN CELL LINE OVCAR-8
45. HUMAN OVARIAN CELL LINE SK-OV-3
46. HUMAN PROSTATE CELL LINE DU145
47. HUMAN PROSTATE CELL LINE PC-3
48. HUMAN RENAL CELL LINE 786-0
49. HUMAN RENAL CELL LINE A498
50. HUMAN RENAL CELL LINE ACHN
51. HUMAN RENAL CELL LINE CAKI-1
52. HUMAN RENAL CELL LINE RXF-393
53. HUMAN RENAL CELL LINE TK-10
