# Supplementary material for: Secondary Analysis of the NCI-60 Whole Exome Sequencing Data Indicates Significant Presence of Propionibacterium acnes Genomic Material in Leukemia (RPMI-8226) and Central Nervous System (SF-295, SF-539, and SNB-19) Cell Lines
Source: PLoS One. 2015 Jun 3;10(6):e0127799. doi: 10.1371/journal.pone.0127799 (PMC4454691; doi:10.1371/journal.pone.0127799)
Supplement: S2 Text — (DOCX) [file pone.0127799.s002.docx]

Appendix 1

Let *m* be a genome size. We will refer to it as *m_h_* for the size of human genome and *m_b_* for the size of bacterial genome. Considering the genome to be double stranded, the number of *n*-mers present in genome of size *m* is 2(*m*-*n*) $\approx$ 2*m* (assuming *m*>>*n*). With 4*^n^* being the total number of different *n*-mers possible, the probability of a given *n*-mer found after a single attempt is $\frac{1}{4^{n}}$ and the probability of a given *n*-mer NOT found after a single attempt is $1-\frac{1}{4^{n}}$. Thus, the probability of a single *n*-mer not to be found after 2*m* attempts (the probability that particular *n*-mer is NOT present in genome of size *m*):

$$\left( 1-\frac{1}{4^{n}} \right)^{2m}$$

The probability of given *n*-mer to be found in genome of size *m* one or more times:

$${1-\left( 1-\frac{1}{4^{n}} \right)}^{2m}$$

The expected number of different *n*-mers present in genome of size *m*:

$$4^{n}\left( {1-\left( 1-\frac{1}{4^{n}} \right)}^{2m} \right)$$

The expected number of *n*-mers from human genome (*m_h_*) found to be simultaneously present in bacterial genome (*m_b_*):

$${2m}_{h}\left( {1-\left( 1-\frac{1}{4^{n}} \right)}^{2m_{b}} \right)$$

The proportion of *n*-mers present in human genome and bacteria simultaneously:

$$\frac{{2m}_{h}\left( {1-\left( 1-\frac{1}{4^{n}} \right)}^{2m_{b}} \right)}{4^{n}\left( {1-\left( 1-\frac{1}{4^{n}} \right)}^{2m_{h}} \right)}$$

For *N* reads taken randomly from the human genome (number of reads in the sequencing dataset) the expected number of reads to be present simultaneously in human and bacteria:

$$N\frac{2m_{h}\left( {1-\left( 1-\frac{1}{4^{n}} \right)}^{2m_{b}} \right)}{4^{n}\left( {1-\left( 1-\frac{1}{4^{n}} \right)}^{2m_{h}} \right)}$$

Using first two terms of the Taylor series approximation:

$${(1-\alpha)}^{x}=1-x\alpha+\frac{x\left( x-1 \right)}{2!}\alpha^{2}-\frac{x\left( x-1 \right)\left( x-2 \right)}{3!}\alpha^{3}+\ldots$$

This formula can be transformed to:

$$N\frac{{2m}_{h}\left( {1-\left( 1-\frac{1}{4^{n}} \right)}^{2m_{b}} \right)}{4^{n}\left( {1-\left( 1-\frac{1}{4^{n}} \right)}^{2m_{h}} \right)}\approx N\frac{2m_{h}\left( 1-1+\frac{2m_{b}}{4^{n}} \right)}{4^{n}\left( 1-1+\frac{2m_{h}}{4^{n}} \right)}=N\frac{2m_{b}}{4^{n}}$$

So, for *N*=30,000,000; *m_h_*=6,000,000,000; *m_b_*=5,000,000 and *n*=32, this value (the expected number of reads to be present simultaneously in human and bacteria) is 1.63x10^-5^. For *n*=80 the value will be 2.05x10^-34^.

Appendix 2

Let *m* be the size of a genome. We will refer to it as *m_h_* for the size of human genome and *m_b_* for the size of bacterial genome. Let *n* be a reads length; *Z* - a proportion of exome in human genome to be enriched during the sample preparation process; *E* - an enrichment coefficient (the proportion of exome/not-exome sequences as a result of enrichment); and *K* - the number of copies of human genomes in the sample. Assuming that the “original” sample has *X* copies of bacteria per human cell, the total composition of reads in the sample can be represented as the sum of three components:

$$\mathrm{KZ}m_{h}+ K\left( 1-Z \right)m_{h}+\mathrm{KX}m_{b}$$

Where $\mathrm{KZ}m_{h}$ are reads deriving from exome sequences; $K\left( 1-Z \right)m_{h}$: reads deriving from the rest (not exome) of the human genome; and $\mathrm{KX}m_{b}$ represents the amount of reads from bacterial genomes.

The enrichment process can be modeled by multiplying the amount (proportion) of exome sequences by the enrichment coefficient, when the rest of the components of the genomic composition of the sample remain constant. Note that the same effect can be reached by dividing everything that is not exome by the enrichment coefficient. Thus, the new composition of the sample will be:

$$\mathrm{EKZ}m_{h}+ K\left( 1-Z \right)m_{h}+\mathrm{KX}m_{b}$$

The observed proportion (fraction) of the bacterial reads in the sample

$$O=\frac{\mathrm{KX}m_{b}}{\mathrm{EKZ}m_{h}+ K\left( 1-Z \right)m_{h}}=\frac{Xm_{b}}{\mathrm{EZ}m_{h}+ \left( 1-Z \right)m_{h}}$$

Which allows for calculating the “original” sample as having *X* copies of bacteria per each human cell:

$$X=\frac{O(EZm_{h}+ \left( 1-Z \right)m_{h})}{m_{b}}$$

So, for O = 2,000/30,000,000; *m_h_*=6,000,000,000; *m_b_*=5,000,000; Z=0.05 (5% of genome enriched); and *E*=20 (enrichment coefficient) the resulting value is equal to 0.156 bacteria per human cell.
